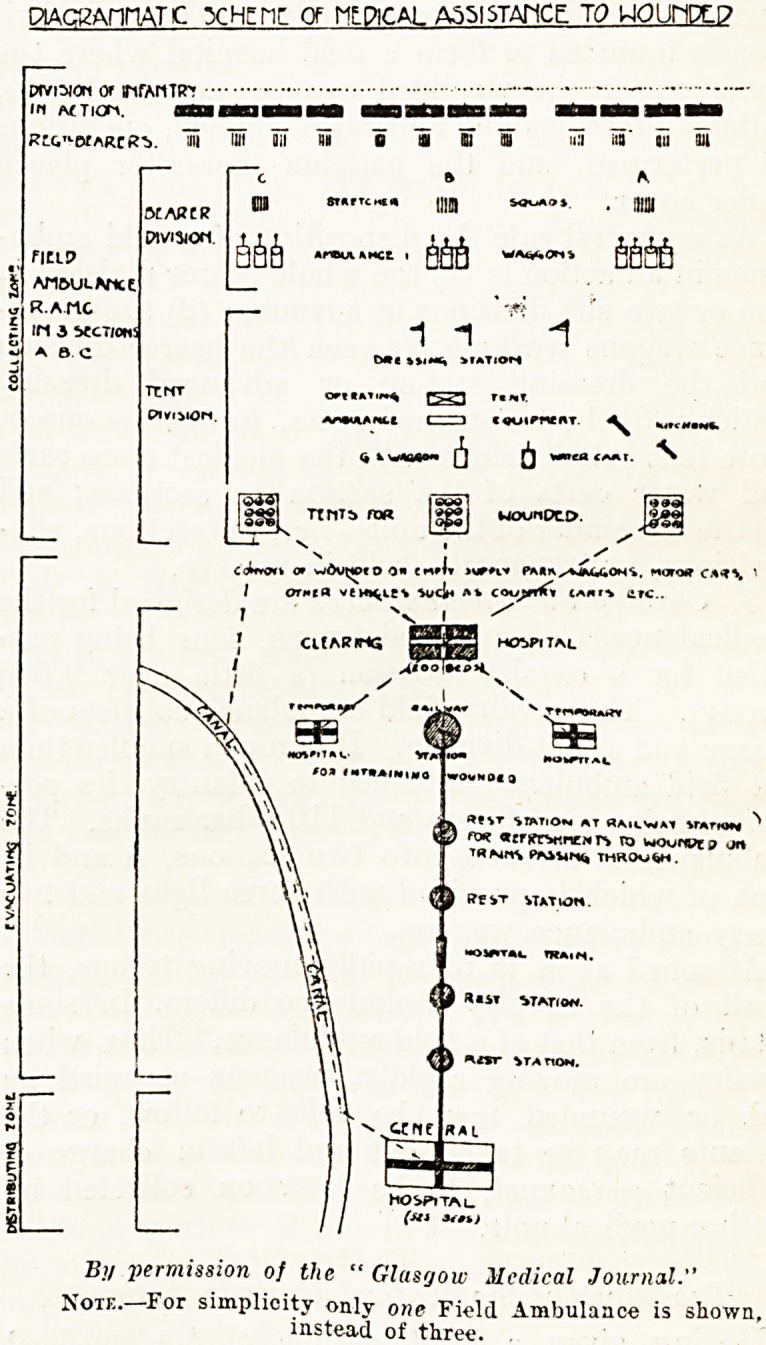# Medical Aid for Soldiers in the Field

**Published:** 1912-11-02

**Authors:** 


### MEDICAL AID FOR SOLDIERS IN THE FIELD.

### Its Principles and Organisation for a Field Force.

THE medical organisation for a field force must, like the troops it is designed to serve, be mobile. This is the first essential point. The second, arising out of the first, is that the sick and wounded are only temporarily provided for. They must be got rid of at the earliest possible moment, as unless this be done the mobility of the medical field unit is at once hampered if not actually annulled.

An army in the field consists of two parts. There are (1) the fully mobile field units which will actually come into touch with the enemy; (2) those forming the "lines of communication" between the field units and the base. Corresponding with this arrangement the theatre of war is divided into three "zones of medical service." These divisions respectively are termed the "collecting," the "evacuating," and the "distributing" zones. Their organisation and relation to the combatant troops may best be understood in tabular form:

| Combatant Troops | Zone | Medical Organisation |
|---|---|---|
| Mobile field units | Collecting | Regimental medical establishments<br>Field ambulances<br>Cavalry field ambulances |
| Lines of communication | Evacuating | Clearing hospi t<br>Ambulance trains<br>Advanced depôts of medical stores<br>General and stationary hospitals (sometimes) |
| Base, including a portion of the lines of communication | Distributing | Stationary and general hospitals<br>Convalescent depôts<br>Ambulance trains<br>Base depôts of medical stores<br>Hospital ships<br>Military hospitals in the United Kingdom |

### COLLECTING ZONE.

In this zone the sick and wounded are collected, appropriate "first aid" is administered, and they are then sent back (if unable immediately to rejoin their units) to the evacuating zone. The medical organisations in the former zone are (1) Regimental Medical Establishments, and (2) and (3) Field Ambulances, for infantry and for cavalry.

1. *Regimental Medical Establishments* are formed by each field unit. A certain number of men belonging to the regiment (in the infantry, bandsmen) are detailed as stretcher-bearers, usually sixteen in all. These work under the direction of the regimental medical officer, assisted by two orderlies supplied with a medical equipment cart.

The regimental medical establishment of a cavalry regiment does not include stretcher-bearers, but men trained in first aid. It is recommended that, as cavalrymen are employed so frequently beyond the reach of professional medical assistance, they should all have some knowledge of first aid. Apart from stretcher-bearers, the organisation of the cavalry regimental medical establishment corresponds pretty closely with that of the infantry.

2. *Field Ambulances* are formed by the R.A.M.C., and are designed for the immediate medical assistance of the troops. Three field ambulances are provided for each division of an army (a little over 18,000 officers and men). The field ambulance is *the* medical field unit. Its details of organisation require some notice.

The ambulance consists of a "bearer division," for the early medical assistance and collection of

DIAGRAMMATIC SCHEME OF MEDICAL ASSISTANCE TO WOUNDED

*By permission of the "Glasgow Medical Journal."*

NOTE.—For simplicity only *one* Field Ambulance is shown, instead of three.

wounded, and a "tent division," for their reception, temporary treatment, and care. It is divisible into three sections, A, B, and C, each capable of acting independently. Each section is organised as a bearer sub-division and a tent sub-division. The transport consists of ambulance wagons (10 in number) for the carriage of sick and wounded, and transport wagons and carts for the carriage of medical and surgical stores, equipment, and water. The *personnel* of the ambulance consists of 10 officers and 241 other ranks.

The officers of the ambulance are as follows: Nine medical officers, and one quartermaster. There are one lieutenant-colonel, commanding the ambulance; two majors; and six captains or subalterns. In each section there are three officers, section A being under the command of the lieutenant-colonel, and sections B and C each under a major. The quartermaster is attached to A section. In each section one of the junior officers is detailed to the bearer sub-division: the tent sub-division is under the charge of the officer commanding the section, assisted by the remaining junior officer of the section. Each tent sub-division is intended to take charge of and accommodate fifty patients.

The bearer division of the ambulance is made up of stretcher-bearers, and each squad of bearers is supplied with restoratives and other means of rendering immediate aid to wounded. The tent division is intended to form a field hospital where the condition of the wounded can be examined into, suitable dressings and apparatus applied, operations be performed, and the patients thereafter placed under cover.

As a general rule the disposition of a field ambulance in an action is (1) the whole bearer division or one or two sub-divisions in advance; (2) the ambulance wagons working between the bearer division and the dressing station or advanced dressing station; (3) the last-named posts, formed by one or more tent sub-divisions with the medical store carts and water carts of the section or sections; and (4) the remainder of the unit—*i.e.*, the sections, sub-divisions, or transport—held in reserve.

3. *Cavalry Field Ambulances* are designed for the medical needs of mounted troops, four being provided for a cavalry division (a little over 9,000 strong). The cavalry field ambulance consists of a bearer and a tent division. It is much smaller than the field ambulance attached to infantry, its *personnel* being six officers and 110 other ranks. The ambulance is divisible into two sections, A and B, each of which is provided with three light and two heavy ambulance wagons.

Attached as it is to rapidly moving troops, the work of the cavalry ambulance differs, in some details, from that of a field ambulance. Thus, when cavalry are moving rapidly, wagons occupied by sick or wounded may be left to follow, or the patients may be taken out and left in charge of sufficient *personnel*, to be later on collected by another medical unit.

*The Work of the Medical Units in Action.*

Having given a brief outline of the medical organisations in the collecting zone, we are now able to indicate the method in which they are used in action.

*Regimental Medical Establishment.*—The regimental stretcher-bearers remain with the regiment to which they belong, and their duty during the engagement is to render first aid to the wounded and to remove them, if possible, from the firing line and place them under cover. Serious cases should, if possible, first be attended to, and the first-aid treatment is confined to the arrest of hæmorrhage, the application of a dressing, the relief of pain by the administration of morphia, and the application of splints, etc., to a broken limb. The seriously wounded should be left where they lie, immediate treatment having been rendered, until the progress of the action allows of systematic removal taking place. A tally or label is attached to each wounded man. For severely wounded a red, and for others a white tally is used. On the tally are entered the nature of the wound, also whether or not an opiate has been given. In addition, the man's name and regimental number, rank, and regiment are entered.

*A Regimental Aid Post* should be selected by the medical officer. The post should be under cover, or out of the line of fire, and should be readily accessible. To it the regimental bearers carry the wounded. The latter are then attended to and left until taken over by the field ambulances.

The regimental officer of a cavalry regimental medical establishment has at his disposal one or more light ambulance wagons, which he uses, if possible, for collecting the wounded. If it be undesirable to use these wagons, first-aid men are despatched mounted, with instructions to use improvised methods of conveyance, either on a horse or on improvised stretchers.

*The Field Ambulance in Action.*

The officer commanding the ambulance unit acts on the principle of not at first employing more sections than are absolutely necessary. In this way he should be able to keep something in hand to meet any development which may arise.

Slightly wounded men, able to walk, are collected, treated, fed, and rested at the *Divisional Collecting Station.* Wherever the station be formed (village, etc.), it should be well to the rear and not on the main line of evacuation. It may be attached to a selected dressing station, or beside reserve sections. From the collecting station the wounded, after being rested, return to their units or are evacuated to the rear.

When the field ambulance has reached the position assigned to it, the bearer division, or sub-divisions, are disengaged, and formed up ready to advance. The stretcher squads will be accompanied, if possible, by the ambulance wagons of the sections to which they belong. If a dressing station is to be opened at this point, a dressing-station party is formed from the tent sub-divisions; but one tent sub-division, with necessary store and water carts, is held in readiness to advance with the bearer division, so as to form an *advanced* dressing station. A dressing station should not be opened too early, and especially if the enemy is falling back, as it is important that it should not get out of touch with the troops. As a rule, however, dressing stations are opened as soon as the troops in the area engage. Dressing stations should not be opened far in advance unless the enemy has commenced to retreat.

The *Bearer Division*, having been formed up, proceeds to establish communication with the regimental medical establishments of the troops engaged. It ascertains from them where the wounded are collected, and it takes them over. It may advance under cover into an area subjected to the enemy's fire; but its main work takes place after the battle. The squads work systematically over the ground, paying special attention in their search to places affording concealment and cover. They search not only for the wounded, collected and dressed by the regimental medical establishments, but also for wounded who have not previously received attention. These latter receive first aid from the stretcher squads, who then attach to each a tally. The whole are then conveyed on stretchers to the dressing station, or to the ambulance wagons, which will have been advanced as near as possible to the area of fighting. Each wagon, after being loaded, moves off to the dressing station, where it is unloaded and returns to the front.

*Dressing Stations* will be prepared at selected points. Use should be made of any available suitable buildings, failing which tents will be pitched. A dressing station should be as far forward as is consistent with reasonable safety, accessible to wheeled vehicles, near but not on a main road, and near water.

The medical store cart is unpacked, medical and surgical equipment taken out, field kitchens constructed, and full provision made for the treatment and feeding of the wounded. This includes preparing the operating outfit, although no cases are operated upon except they be urgent.

Considerable organising skill is called for at a dressing station, in the formation of separate departments for receiving, recording, and classifying the wounded; for severe cases; for slight cases; and for the dying. Kitchens, latrines, refuse disposal, etc., all require to be arranged for.

*Disposal of Wounded from Dressing Station.*

The wounded are sent back from the dressing station to the clearing hospital, the orders for the disposal of the wounded indicating the point where the clearing hospital is established. Those wounded who are able to walk are first sent back; next, those able to travel sitting up; and, lastly, the lying-down cases are conveyed in wagons. The supply wagons for the troops are usually employed for this purpose when returning empty to the rear. In addition, it may be possible to procure transport in country carts. If not possible at once to move up a clearing hospital to relieve the dressing stations, these are kept open, and they remain immobile till the wounded have been evacuated, after which the tent sub-divisions may be advanced. In any circumstances, the bearer divisions always remain mobile, and are available to proceed with the troops.

### EVACUATING ZONE.

We now pass on to consider the organisations in the evacuating zone. First of all comes the unit to which several references have been made in the immediately preceding paragraphs, viz:—

*The Clearing Hospital.*

The function of the clearing hospital is to receive and to pass on the sick and wounded collected by the ambulances. Clearing hospitals are organised in the proportion of one to each division (18,000 strong) of the field army. Each clearing hospital is designed to take charge of 200 patients; but it is capable of being expanded to take in many more. Its *personnel* consists of eight officers and seventy-seven other ranks. While usually situated at an advanced base, a clearing hospital may, if many sick or wounded have to be dealt with, be advanced close up to the division. Again, if the division is to advance, the clearing hospital may be sent up to the ambulances to take over their wounded, and allow the ambulances to go on with the division.

If situated at an advanced base, the clearing hospital will receive the wounded brought back from the ambulances in empty supply wagons. If, on account of large numbers of sick and wounded, it has been moved up close to the division, it will receive wounded direct from the wagons of the field ambulances. It may have to organise *Rest Stations* on the lines of communication, with transport moving between them, in this way forming a line of evacuation for the wounded. If this is required, the *personnel* will be told off as rest-station parties, and as a main body. The *personnel* of a rest station consists of an officer with one or more sergeants and four or five rank and file. They are equipped with cooking utensils, medical comforts, etc.

The clearing hospital and its detachments forming rest stations take over existing buildings, and prepare them for the temporary reception of patients. Smaller detachments may form rest stations at railway stations or along the line of route of road or water transport. These are for the preparation of food or restoratives; but they will be ready to receive serious cases unable to proceed further.

As in the field ambulances, the officer commanding the clearing hospital must take care that it does not become "clogged." He must therefore see that the wounded are passed on whenever possible. If any of the patients are fit for duty at the front, they will be sent forward to rejoin their units.

*Ambulance Trains.*

Ambulance trains are organised to carry 100 sick lying down, and are in the proportion of one to each division of the army. The *personnel* of a train is two officers and eighteen other ranks. While the train usually receives the wounded from the clearing hospital, it may, if the situation permits, be in direct touch with the field ambulances.

*Advanced Depôts of Medical Stores* are formed, in the proportion of one to every two divisions, at the advanced base. They replenish field medical units and units on the lines of communication with medical and surgical equipment.

The wounded taken over by the ambulance train are conveyed by it out of the evacuating into the distributing zone. Here they are taken into one of the base hospitals, whence they are, after recovery, sent back to the front or invalided home. The organisation of the distributing zone does not come at present within our purview. We have endeavoured merely to give the reader an outline of medical organisation in the field, but we trust that by means of the explanatory plan it has been made sufficiently clear.

## Figures and Tables

**Figure f1:**